# N^pro^ of classical swine fever virus contributes to pathogenicity in pigs by preventing type I interferon induction at local replication sites

**DOI:** 10.1186/1297-9716-45-47

**Published:** 2014-04-17

**Authors:** Tomokazu Tamura, Naofumi Nagashima, Nicolas Ruggli, Artur Summerfield, Hiroshi Kida, Yoshihiro Sakoda

**Affiliations:** 1 Laboratory of Microbiology, Department of Disease Control, Graduate School of Veterinary Medicine, Hokkaido University, Sapporo 060-0818, Japan; 2 Institute of Virology and Immunology, Sensemattstrasse 293, CH-3147 Mittelhäusern, Switzerland; 3 Research Center for Zoonosis Control, Hokkaido University, Sapporo 001-0020, Japan

## Abstract

Classical swine fever (CSF) caused by CSF virus (CSFV) is a highly contagious disease of pigs. The viral protein N^pro^ of CSFV interferes with alpha- and beta-interferon (IFN-α/β) induction by promoting the degradation of interferon regulatory factor 3 (IRF3). During the establishment of the live attenuated CSF vaccine strain GPE^-^, N^pro^ acquired a mutation that abolished its capacity to bind and degrade IRF3, rendering it unable to prevent IFN-α/β induction. In a previous study, we showed that the GPE^-^ vaccine virus became pathogenic after forced serial passages in pigs, which was attributed to the amino acid substitutions T830A in the viral proteins E2 and V2475A and A2563V in NS4B. Interestingly, during the re-adaptation of the GPE^-^ vaccine virus in pigs, the IRF3-degrading function of N^pro^ was not recovered. Therefore, we examined whether restoring the ability of N^pro^ to block IFN-α/β induction of both the avirulent and moderately virulent GPE^-^-derived virus would enhance pathogenicity in pigs. Viruses carrying the N136D substitution in N^pro^ regained the ability to degrade IRF3 and suppress IFN-α/β induction in vitro. In pigs, functional N^pro^ significantly reduced the local IFN-α mRNA expression in lymphoid organs while it increased quantities of IFN-α/β in the circulation, and enhanced pathogenicity of the moderately virulent virus. In conclusion, the present study demonstrates that functional N^pro^ influences the innate immune response at local sites of virus replication in pigs and contributes to pathogenicity of CSFV in synergy with viral replication.

## Introduction

Classical swine fever (CSF) is an economically important and highly contagious disease of pigs that is caused by CSF virus (CSFV). CSFV belongs to the genus *Pestivirus* of the family *Flaviviridae*, together with bovine viral diarrhea virus and border disease virus [1]. CSFV possesses a single-stranded positive-sense RNA genome of approximately 12.3 kb with one large open reading frame flanked by a 5’ and 3’ untranslated region. It encodes approximately 4000 amino acids that yield at least 12 cleavage products N^pro^, C, E^rns^, E1, E2, p7, NS2, NS3, NS4A, NS4B, NS5A, NS5B through co- and post-translational processing of the polyprotein by cellular and viral proteases [1,2]. The structural components of the CSFV virion include the capsid (C) protein and the glycoproteins E^rns^, E1 and E2. The non-structural proteins NS3 through NS5B are essential for pestivirus RNA replication, whereas N^pro^, p7, NS2 and all of the structural proteins are dispensable [1]. Recently, alternative cleavage products of NS3 were identified [3].

N^pro^, a protein unique to pestiviruses, autocatalytically generates its own carboxy terminus through its protease activity [1]. N^pro^ per se is dispensable for pestivirus replication [4], but exerts accessory functions by interfering with innate immune activation. N^pro^ interacts with and mediates the degradation of interferon regulatory factor 3 (IRF3), blocking alpha- and beta-interferon (IFN-α/β) induction in most target cells [5,6]. In plasmacytoid dendritic cells (pDCs), N^pro^ interacts with interferon regulatory factor 7 (IRF7) without inducing IRF7 degradation, dampening IRF7-dependent IFN-α induction by yet unknown mechanisms [7]. The C-terminal half of N^pro^ harbors a zinc-binding TRASH domain consisting of C112-X21-C134-X3-C138 that is required for the interaction with these factors [7-9]. By means of N^pro^, pestiviruses interfere with IFN-α/β induction, which can be measured with strong viral IFN-α/β inducers such as Newcastle disease virus (NDV) [10,11]. This is termed “exaltation of NDV” (END), referring to the observation that NDV replicates more efficiently in cells infected with pestivirus than in absence of pestivirus infection [11]. During the generation of the live attenuated vaccine strain GPE^-^ from the parental ALD strain, the N^pro^-mediated suppression of IFN-α/β induction was lost, resulting in a virus that did not exhibit the END phenomenon [12,13]. This biotype was designated END-negative (END^-^) CSFV. In a previous recent study from our laboratory, the live-attenuated CSF vaccine strain GPE^-^ was re-adapted to its natural host by multiple serial passages in pigs in order to select for increased replication efficiency and pathogenicity, with the aim of identifying determinants of CSFV virulence. The GPE^-^ virus became pathogenic after 11 serial passages in pigs by intramuscular injection of tonsil homogenates. Three amino acid substitutions, T830A in E2 and V2475A and A2563V in NS4B, were found to be responsible for the acquisition of pathogenicity [14]. Interestingly, during the serial passages of the GPE^-^ vaccine virus in pigs, the IRF3-degrading function of N^pro^ was not recovered.

In a previous study, mutations designed to knock out N^pro^-mediated IRF3 degradation did not alter the pathogenicity of the highly virulent CSFV strain Eystrup and caused only slight attenuation of the moderately virulent Alfort/187 virus [10]. Here, we examined whether restoring the N^pro^ function of an END^-^ virus would enhance its pathogenicity. To this end, viruses with a single amino acid substitution restoring N^pro^-mediated IRF3 degradation in the GPE^-^ vaccine virus backbone and in a GPE^-^-derived moderately virulent mutant [14] were generated. The pathogenicity and innate immune responses to infection with the parental and mutant viruses were investigated by experimental infection of pigs.

## Materials and methods

### Cells

The swine kidney cell line SK-L [15] was propagated in Eagle’s minimum essential medium (MEM) (Nissui Pharmaceutical, Tokyo, Japan) supplemented with 0.295% tryptose phosphate broth (Becton Dickinson, San Jose, CA, USA), 10 mM N,N-bis-(2-hydroxyethyl)-2-aminoethanesulfonic acid (Sigma-Aldrich, St. Louis, MO, USA) and 10% horse serum (Life Technologies, Carlsbad, CA, USA). The SK6-MxLuc cell line carrying a Mx/Luc reporter gene [16] was propagated in MEM supplemented with 0.295% tryptose phosphate broth and 7% horse serum. The human embryonic kidney cell line 293T was maintained in Dulbecco’s MEM (Life Technologies) and 10% fetal calf serum (Cambrex, Grand Island, NY, USA). All cells were incubated at 37 °C in the presence of 5% CO_2_.

### Viruses

The CSFV vGPE^-^, vGPE^-^/T830A; V2475A; A2563V and vEy-37 were derived from the full-length cDNA clones pGPE^-^, pGPE^-^/T830A; V2475A; A2563V [14] and pEy-37 [17], respectively. The vGPE^-^- and vGPE^-^/T830A; V2475A; A2563V-derived viruses with the N136D mutation in N^pro^ (vGPE^-^/N136D and vGPE^-^/N136D; T830A; V2475A; A2563V) were rescued from mutant cDNA plasmids that were constructed using the QuickChange XL Site-Directed Mutagenesis Kit (Agilent Technologies, Santa Clara, CA, USA) and oligonucleotide primers containing the respective mutation, applying standard techniques described previously [14]. All cDNA-derived viruses were rescued as described previously [18]. In brief, plasmid constructs were linearized at the *Srf*I site located at the end of the viral genomic cDNA sequence, and RNA was obtained by run-off transcription using the MEGAscript T7 kit (Life Technologies). After DNase I treatment and purification on S-400 HR Sephadex columns (GE Healthcare, Buckinghamshire, UK), RNA was quantified using a spectrophotometer and used to electroporate SK-L cells. The complete genomes of the rescued viruses were verified by nucleotide sequencing to exclude any accidental mutation. The rescued viruses were stored at -80 °C.

### Sequencing

The full-length cDNA clones and in vitro-rescued viruses were completely sequenced as described previously [14]. In brief, nucleotide sequencing of cDNA clones and PCR fragments from viral RNA was performed using the BigDye Terminator v3.1 Cycle Sequencing Kit (Life Technologies) and a 3500 Genetic Analyzer (Life Technologies). Sequencing data were analyzed using GENETYX®-Mac version 13 software (GENETYX, Tokyo, Japan).

### Virus titration

Virus titers were determined by end-point dilution on SK-L cells in 96-well plates and immunoperoxidase staining using the anti-NS3 mAb 46/1 as described previously [19,20]. The titers were calculated using the formula of Reed and Muench [21] and expressed in 50% tissue culture infective dose (TCID_50_) per mL or gram.

### SDS-PAGE and Western blot analysis

SDS-PAGE and Western blotting were performed as described previously [22]. The concentration of the SDS polyacrylamide gels was 7.5%. The anti-porcine IRF3 mAb 34/1 [6] was used as primary antibody. Signals were detected with the Immobilon Western Detection Reagents (Millipore, Bedford, MA, USA) and the LumiVision PRO 400EX system (Aisin Seiki, Aichi, Japan) and quantified using the LumiVision Analyzer 2.0 software (Aisin Seiki).

### Virus replication kinetics

The replication kinetics of the parental and mutant viruses in SK-L cells were determined by inoculation of confluent cell monolayers at a multiplicity of infection (MOI) of 0.1 TCID_50_ and collection of cell culture supernatants at different times post-inoculation (pi). After inoculation, the SK-L cells were incubated at 37 °C in the presence of 5% CO_2_. The supernatants were collected at 0, 16, 24, 48, 72, 96, 120, 144 and 168 hours post-inoculation (hpi). The virus titers were determined in duplicate using the SK-L cells. Statistically significant differences were detected using the Student’s *t* test.

### IFN bioassay

The bioactivity of porcine IFN-α/β was assayed using the Mx/Luc reporter gene assay as previously described [16]. Viruses present in culture supernatants and in porcine serum samples were inactivated using a UV crosslinker (ATTO, Tokyo, Japan). SK6-MxLuc cells were seeded in 24-well plates at a density of 1.5 × 10^5^ cells per well. After 20 h, 400 μL of samples diluted appropriately to allow measurement in the linear range of the assay or a porcine IFN-β standard were added to the cells which were then incubated at 37 °C in the presence of 5% CO_2_. Recombinant porcine IFN-β produced in 293T cells was used as a standard. The cell extracts were prepared with 100 μL of passive lysis buffer, and firefly luciferase activities were measured using a dual luciferase reporter assay system (Promega, Madison, WI, USA) and a PowerScan4 microplate reader (DS Pharma Biomedical, Osaka, Japan). The data were analyzed using Gen5 software (DS Pharma Biomedical). Results were recorded for three independent experiments, and each experiment was performed in duplicate. Statistically significant differences were detected using the Student’s *t* test. Virus inactivation was monitored on SK-L cells using immunohistochemistry with mAb 46/1.

### Experimental infection of pigs

In order to assess the pathogenicity of the cDNA-derived viruses, groups of eight 4-week-old crossbred Landrace × Duroc × Yorkshire SPF pigs (Yamanaka Chikusan, Hokkaido, Japan) were injected intramuscularly with 1 mL of virus from cell culture supernatant. For each group, 5 pigs were kept for 14 days and 3 pigs for 5 days, and clinical signs monitored daily. With the 5 pigs kept for 14 days, blood was collected in tubes containing EDTA (Terumo, Tokyo, Japan) on days 0, 3, 5, 7, 9, 11 and 14 pi. Total leucocytes and platelets were counted using a pocH-100iV Diff apparatus (Sysmex, Hyogo, Japan). The pigs that survived the infection were euthanized on day 14 pi, and tissues from brain, tonsils, spleen, kidneys, adrenal glands, mesenteric lymph nodes and colon were collected aseptically. The collected samples were homogenized in MEM to obtain a 10% suspension for virus isolation. Virus isolation was performed by inoculation of samples into SK-L monolayer of 6-well plates. As for virus antigen-positive samples, virus titration was conducted and their titers were expressed as TCID_50_ per mL (blood) or gram (tissue). With the 3 pigs kept for 5 days, whole blood was collected for serum preparation at 0, 12, 24, 36, 48, 72, 96 and 120 hpi. These 3 pigs were euthanized at 120 hpi, and tissues from tonsils, spleen and mesenteric lymph nodes were collected for quantitative measurement of mRNA expression.

All animal experiments were performed in self-contained isolator units (Tokiwa Kagaku, Tokyo, Japan) in a BSL-3 facility of the Graduate School of Veterinary Medicine, Hokkaido University, Sapporo, Japan at the serial time points. The institutional animal care and use committee of the Graduate School of Veterinary Medicine authorized this animal experiment (approval numbers: 10–0106 and 12–0013), and all experiments were performed according to the guidelines of this committee.

### Quantitative real-time PCR (qPCR)

For quantification of IFN-α gene expression, total RNA of tissues from tonsils, spleen and mesenteric lymph nodes was extracted using the RNeasy Mini Kit (Qiagen, Hiden, Germany) according to the manufacturer’s protocol. Contaminant genomic DNA was removed by DNase I treatment using a RNase-Free DNase set (Qiagen). Total RNA was quantified and cDNA synthesized using Random Primers (N)_9_ (Takara Bio, Shiga, Japan) and SuperScript III Reverse Transcriptase (Life Technologies) in 20 μL reaction mixtures containing 1 μg of total RNA. The cDNA was analyzed by qPCR using the KAPA PROBE Fast qPCR Kit (Kapa Biosystem, Woburn, MA, USA) and a Light Cycler® 480 System II (Roche, Basel, Switzerland). The primers and cycling conditions reported by Lee et al. [23] were used for IFN-α detection. The gene expression data were normalized by assessing mRNA abundance of the housekeeping gene glyceraldehyde 3-phosphate dehydrogenase (GAPDH). GAPDH gene expression was quantified by qPCR according to a previous report [24]. Quantitative values were calculated using the mean of duplicated results. The statistically significant differences were determined using the Student’s *t* test.

## Results

### The amino acid substitution N136D in N^pro^ of the GPE^-^-derived viruses restores N^pro^-mediated IRF3 degradation

**Figure 1:**
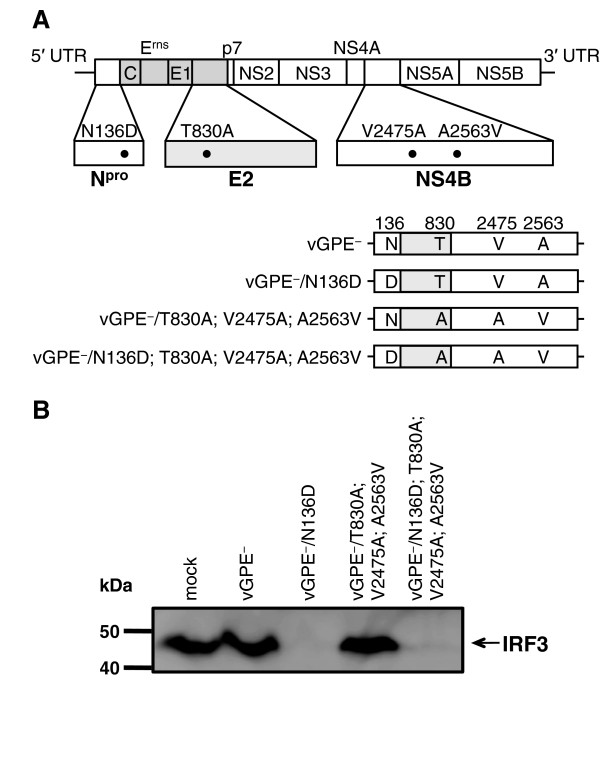
**Effect of restoring the zinc-binding domain in N**^**pro **^**of GPE**^**-**^**-derived viruses on IRF3 degradation. (A)** The amino acid substitution N136D was introduced in N^pro^ of vGPE^-^ and vGPE^-^/T830A; V2475A; A2563V by site-directed mutagenesis. **(B)** Swine kidney cells SK-L were inoculated at an MOI of 1 TCID_50_ per cell with the parent and mutant viruses as indicated. The concentration of the SDS polyacrylamide gels was 7.5%. Proteins were detected at 96 hpi by Western blotting analysis using the anti-porcine IRF3 mAb.

Previous studies demonstrated that Cys112, Cys134, Asp136 and Cys138 in N^pro^ of CSFV form a zinc-binding domain and are essential for the interaction of N^pro^ with IRF3 and inducing proteasomal degradation of IRF3 [8-10]. The GPE^-^ virus encodes an asparagine instead of an aspartic acid at position 136 of the zinc-binding domain [10,25]. It was shown previously that this substitution is sufficient to abolish the IRF3 degrading function of N^pro^ completely [10]. As expected, substitution of asparagine with aspartic acid at position 136 (N136D) in N^pro^ of the END^-^ vGPE^-^ virus or of the triple mutant virus vGPE^-^/T830A; V2475A; A2563V restored the capacity of the virus to bind and degrade IRF3. Infection of SK-L cells with the vGPE^-^/N136D and vGPE^-^/N136D; T830A; V2475A; A2563V, resulted in degradation of IRF3 to undetectable levels after 96 h (Figure 1). Accordingly, the GPE^-^-derived viruses carrying the mutation in N^pro^ regained the capacity to cause the END phenomenon in swine cells (data not shown).

### Restoring N^pro^-mediated IRF3 degradation in the vGPE^-^ and GPE^-^-derived triple mutant virus results in suppression of IFN-α/β production and affects virus production in cell culture

**Figure 2:**
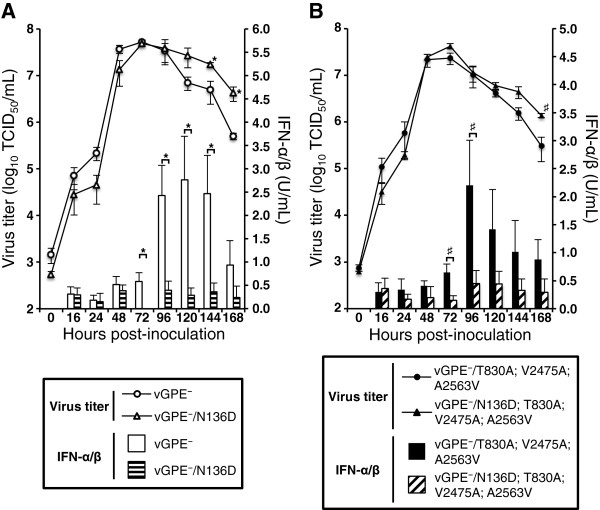
**Growth kinetics and IFN-α/β production after infection of swine cells with the GPE**^**-**^**-derived viruses.** Swine SK-L cells were inoculated at an MOI of 0.1 TCID_50_ per cell with the indicated viruses and incubated at 37 °C in the presence of 5% CO_2_. The supernatants were collected at 0, 16, 24, 48, 72, 96, 120, 144 and 168 hpi. Virus titers were determined in duplicate in SK-L cells and expressed as TCID_50_/mL, and IFN-α/β bioactivity was measured in duplicate using the SK6-MxLuc reporter cells. These data were compared with vGPE^-^ and vGPE^-^/N136D **(A)**, and with vGPE^-^/T830A; V2475A; A2563V and vGPE^-^/N136D; T830A; V2475A; A2563V **(B)**. Each curve and bar presents the mean from three independent infections, with error bars showing the standard errors. The significance of the differences between was calculated using the Student’s *t* test. * indicates *p* < 0.05 between vGPE^-^ and vGPE^-^/N136D. # indicates *p* < 0.05 between vGPE^-^/T830A; V2475A; A2563V and vGPE^-^/N136D; T830A; V2475A; A2563V.

In order to assess the effect of restoring N^pro^-mediated IRF3 degradation on viral growth kinetics in swine cells, multistep growth curves of viruses carrying either asparagine or aspartic acid at position 136 in N^pro^ were determined in the type I IFN-competent swine SK-L cells. The viruses carrying the single N136D amino acid substitution in N^pro^ (vGPE^-^/N136D and vGPE^-^/N136D; T830A; V2475A; A2563V) showed an initial replication rate similar to that of the parental viruses and reached a peak titer at 72 hpi (Figure 2A and B). After the peak titer was reached, the virus titers of the mutants that suppressed interferon induction tended to be higher than the titers of the corresponding END^-^ viruses. Complete suppression of IFN-α/β production conferred by the N136D substitution was confirmed by measuring IFN-α/β bioactivity in the supernatants of swine SK-L cells infected with the mutant and parental viruses. Taken together, these data suggest that the functional N^pro^ might affect virus production by dampening the innate immune response.

### Restoring N^pro^-mediated inhibition of IFN-α/β induction in the moderately virulent GPE^-^-derived triple mutant virus enhances pathogenicity in pigs

**Figure 3:**
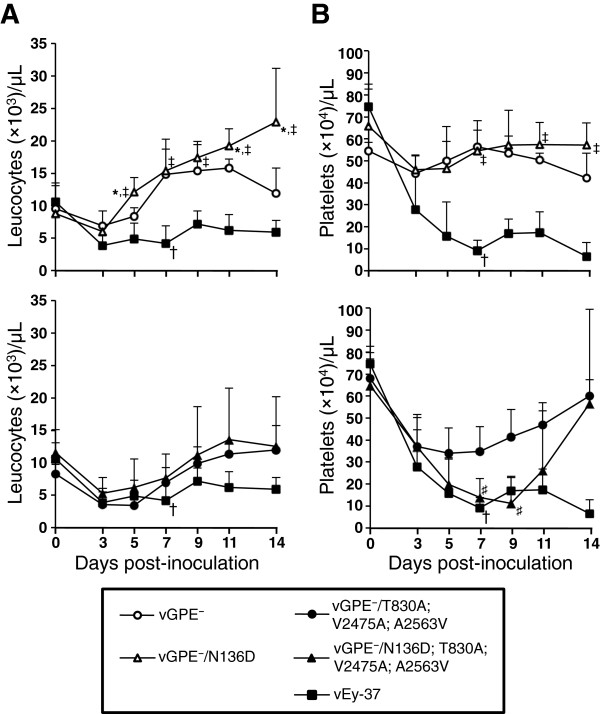
**Leucocyte and platelet counts in pigs inoculated with the GPE**^**-**^**-derived viruses and with vEy-37.** Groups of 5 pigs were inoculated with the indicated viruses and blood was collected on days 0, 3, 5, 7, 9, 11 and 14 pi. The leucocyte **(A)** and platelet **(B)** counts were determined for each time point and are shown as mean values with error bars representing the standard deviation. The statistical significance of the differences between the cell counts was calculated using the Student’s *t* test. * indicates *p* < 0.05 between vGPE^-^ and vGPE^-^/N136D. ‡ indicates *p* < 0.05 between vGPE^-^/N136D and vEy-37. # indicates *p* < 0.05 between vGPE^-^/T830A; V2475A; A2563V and vGPE^-^/N136D; T830A; V2475A; A2563V. Two pigs inoculated with the vEy-37 virus died on day 7 pi (indicated by †). All remaining pigs were euthanized on day 14 pi.

In order to determine the contribution of N^pro^-mediated suppression of IFN-α/β induction to pathogenicity, five 4-week-old pigs were injected intramuscularly with 10^7.0^ TCID_50_ of N136D mutant or parental GPE^-^-derived viruses. As a control, 5 pigs were inoculated with the highly virulent vEy-37 virus. The pigs inoculated with the GPE^-^-derived virus carrying functional N^pro^ (vGPE^-^/N136D) and with the vGPE^-^ parental virus had only a slight transient leukocytopenia and thrombocytopenia (Figure 3A and B, top panels). No clinical symptoms were observed with any of these two viruses (Table 1). Very little or no virus was recovered from blood and tonsils of these pigs (Table 1). All 5 pigs inoculated with the virus carrying the N136D mutation restoring functional N^pro^ along with the three mutations T830A, V2475A and A2563V (vGPE^-^/N136D; T830A; V2475A; A2563V) had severe thrombocytopenia and showed clinical symptoms of CSF such as weight loss, cyanosis and ataxia (Figure 3B bottom panel and Table 1). Two of these pigs had arthritis. Leukocytopenia caused by vGPE^-^/N136D; T830A; V2475A; A2563V infection was similar to that caused by vGPE^-^/T830A; V2475A; A2563V infection (Figure 3A bottom panel). Importantly, virus was detectable in the blood of all pigs as early as day 3 pi and remained detectable for a longer time period than in pigs infected with the triple mutant lacking the N136D substitution (Table 1). High titers of virus were detected in all collected tissues of one pig infected with the vGPE^-^/N136D; T830A; V2475A; A2563V virus. Infectious virus was detected in at least 3 collected tissues of the remaining four pigs. With the tonsils and mesenteric lymph nodes, virus was recovered from all infected pigs. In contrast, in pigs infected with the triple mutant virus carrying non-functional N^pro^ (vGPE^-^/T830A; V2475A; A2563V), virus could be recovered only from tonsils and from mesenteric lymph nodes in 2 of 5 animals (Table 1). With the highly virulent vEy-37 virus, infection resulted in severe leukocytopenia, thrombocytopenia and clinical signs in all 5 pigs, and 2 of 5 pigs died on day 7 pi. High titers of virus were detected in all blood and tissue samples from vEy-37-infected pigs. These data demonstrate that N^pro^-mediated suppression of IFN-α/β induction can prolong virus replication in tissue and enhance pathogenicity of a moderately virulent CSFV but does not render an avirulent virus pathogenic. Nevertheless, restoration of the N^pro^ function in the moderately virulent virus did not enhance pathogenicity to the level of the highly virulent vEy-37 virus.

**Table 1:** Clinical symptoms and virus recovery from pigs inoculated with CSFVs

		**Virus recovery from:**												
**Inoculated virus**	**Clinical symptoms**^**†**^	**Blood (log**_**10 **_**TCID**_**50**_**/mL)**^**‡ **^**on days pi**						**Tissue (log**_**10 **_**TCID**_**50**_**/g)**^**§**^						
		**3**	**5**	**7**	**9**	**11**	**14**	**Brain**	**Tonsils**	**Spleen**	**Kidneys**	**Adrenal glands**	**Mesenteric lymph node**	**Colon**
vGPE^-^	-	-	-	-	-	-	-	-	-	-	-	-	-	-
	-	+	-	-	-	-	-	-	+	-	-	-	-	-
	-	-	-	-	-	-	-	-	+	-	-	-	-	-
	-	-	+	-	-	-	-	-	+	-	-	-	-	-
	-	-	+	-	-	-	-	-	+	-	-	-	-	-
vGPE^-^/N136D	-	+	-	-	-	-	-	-	+	-	-	-	-	-
	-	≤ 1.6	-	-	-	-	-	-	≤ 2.3	-	-	-	-	-
	-	+	-	-	-	-	-	-	≤ 2.1	-	-	-	-	-
	-	-	-	-	-	-	-	-	-	-	-	-	-	-
	-	-	-	-	-	-	-	-	+	-	-	-	-	-
vGPE^-^/T830A; V2475A; A2563V	Cyanosis	≤ 1.0	≤ 2.1	≤ 1.0	≤ 1.0	-	-	-	4.0	-	-	-	-	-
	-	≤ 1.0	2.6	3.6	≤ 2.6	+	-	-	3.3	-	-	-	+	-
	Weight loss, cyanosis	≤1.1	2.8	≤ 2.3	≤ 1.3	-	-	-	3.7	-	-	-	-	-
	Cyanosis	≤ 1.0	2.6	2.0	≤ 1.0	-	-	-	3.0	-	-	-	-	-
	Diarrhea, cyanosis	≤ 2.1	2.3	2.6	3.1	+	-	-	4.1	-	-	-	≤ 2.3	-
vGPE^-^/N136D; T830A; V2475A; A2563V	Weight loss, coarse fur, cyanosis, astasia	2.6	4.8	5.3	5.6	5.1	4.6	4.3	7.0	6.3	5.6	5.8	6.0	5.8
	Weight loss, coarse fur	3.3	4.0	4.3	4.6	4.3	≤ 1.3	-	≤ 3.8	≤ 2.3	-	+	3.0	≤ 2.3
	Astasia, arthritis	3.3	4.0	3.6	+	-	-	-	3.0	≤ 2.1	-	-	≤ 2.0	-
	Weight loss, arthritis	2.8	3.6	2.9	+	-	-	-	4.1	-	3.8	-	≤ 2.0	-
	Weight loss, coarse fur	2.3	4.3	4.0	3.8	+	-	-	4.0	≤ 2.0	≤ 3.1	≤ 2.3	≤ 2.5	-
vEy-37	Fever, cyanosis, weight loss	5.5	5.0	5.8	6.0	5.8	4.6	6.0	6.8	7.6	7.8	6.0	8.0	7.0
	Low-sproted, anorexia, diarrhea, weight loss, cyanosis, death	4.0	6.0	6.6	NT	NT	NT	6.1	7.0	6.6	5.3	6.0	7.0	5.5
	Fever, low-sproted, anorexia, diarrhea, cyanosis, death	5.3	6.1	6.1	NT	NT	NT	5.0	6.6	6.3	5.6	5.3	6.8	5.8
	Fever, anorexia, diarrhea, weight loss, cyanosis	5.3	6.6	6.7	6.0	6.0	5.1	6.0	7.3	6.8	7.6	6.6	7.5	4.6
	Fever, anorexia, diarrhea, weight loss, cyanosis	4.8	6.0	6.0	6.0	6.8	6.6	6.3	7.6	7.8	7.5	6.8	8.0	7.8

^†^-: not observed.

^‡^-: not isolated, NT: not tested, +: isolated in a 6-well plate and was lower detection limit of TCID_50_ (10^0.8^ TCID_50_/mL) in a 96-well plate.

^§^-: not isolated, +: isolated in a 6-well plate and was lower detection limit of TCID_50_ (10^1.8^ TCID_50_/mL) in a 96-well plate.

### Local N^pro^-mediated suppression of IFN-α/β induction by the moderately virulent GPE^-^-derived triple mutant virus carrying functional N^pro^ enhances systemic IFN-α/β responses

**Figure 4:**
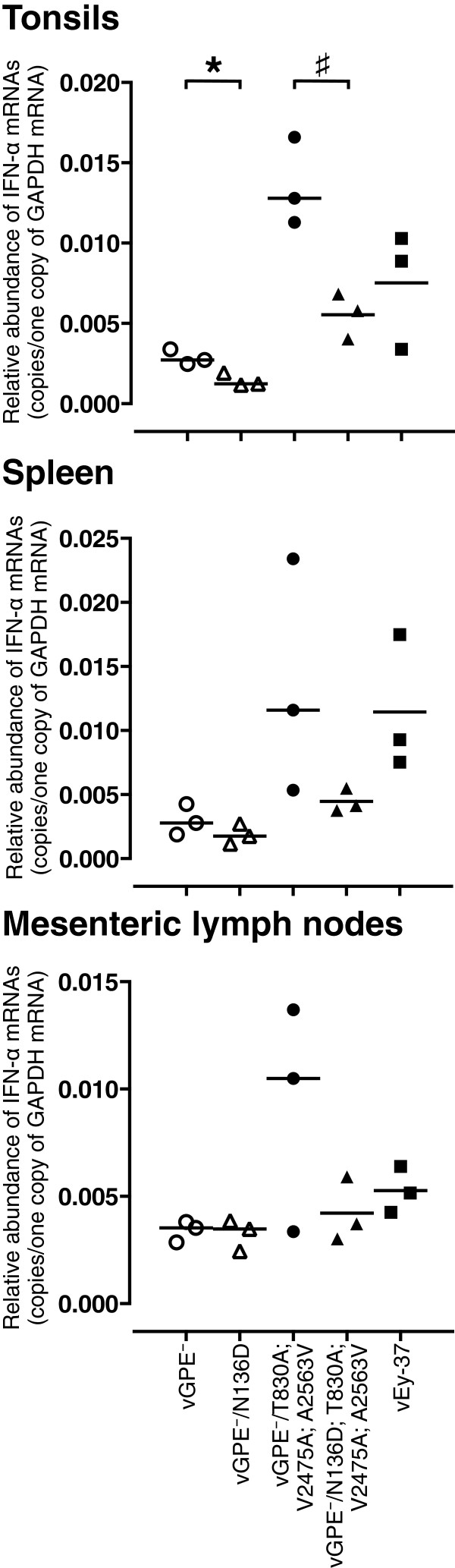
**IFN-α mRNA expression in local immune tissues infected with the GPE**^**-**^**-derived viruses and with vEy-37.** The IFN-α mRNA level of tonsils, spleen and mesenteric lymph nodes collected from 3 pigs per group at 120 hpi was analyzed by qPCR. The mRNA levels are shown as mean value after normalization with GAPDH mRNA content. The mean values with error bars represent the standard error. The statistical significance of the differences between the IFN-α mRNA levels was calculated using the Student’s *t* test. * indicates *p* < 0.05 between vGPE^-^ and vGPE^-^/N136D. # indicates *p* < 0.05 between vGPE^-^/T830A; V2475A; A2563V and vGPE^-^/N136D; T830A; V2475A; A2563V.

**Figure 5:**
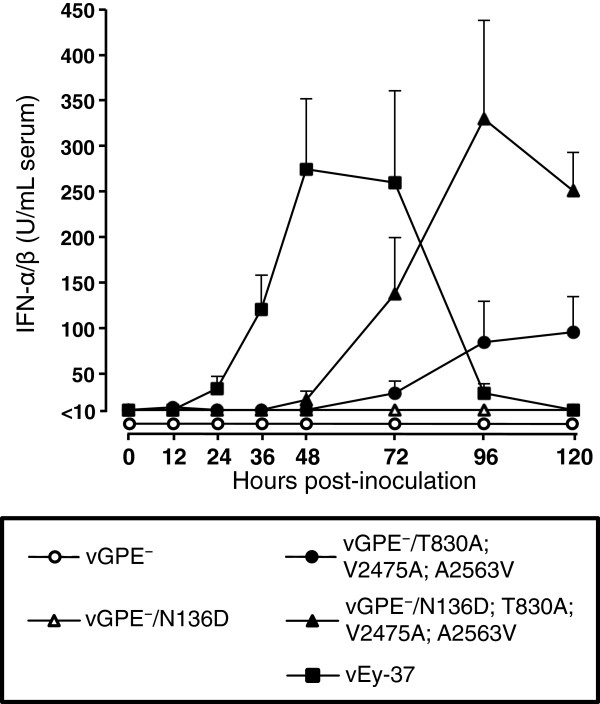
**IFN-α/β bioactivity in serum of pigs inoculated with the vGPE**^**-**^**-derived viruses and with vEy-37.** Groups of 3 pigs were inoculated with the indicated viruses and blood was collected at 0, 12, 24, 36, 48, 72, 96 and 120 hpi. Serum IFN-α/β bioactivity was measured in duplicate using SK6-MxLuc cells. The error bars represent the standard deviation.

We next assessed whether restoring the N^pro^ function with the N136D substitution in the vGPE^-^ parental and triple mutant viruses had any effect on the innate immune responses. To this end, three 4-week-old pigs per group were injected intramuscularly with the same dose of the parental or mutant viruses or with vEy-37 as in the previous experiment. Local and systemic IFN-α levels were measured by mRNA quantification in tonsils, spleen and mesenteric lymph nodes, and by quantification of IFN-α/β bioactivity in the serum, respectively. The IFN-α mRNA expression in the lymphoid tissues was lower with the vGPE^-^/N136D; T830A; V2475A; A2463V virus compared with the parental vGPE^-^/T830A; V2475A; A2563V virus (Figure 4), which is consistent with results obtained in cell culture. This was particularly evident in tonsils. In the serum, no IFN-α/β bioactivity was detected after infection with vGPE^-^ and vGPE^-^/N136D at any time point after infection (Figure 5). Interestingly, significantly higher IFN-α/β bioactivity was observed in sera from pigs inoculated with the vGPE^-^/N136D; T830A; V2475A; A2463V virus carrying functional N^pro^ than in pigs inoculated with the parental vGPE^-^/T830A; V2475A; A2563V virus, despite the N^pro^-mediated suppression of IFN-α/β induction observed in the lymphoid tissues. Infection with the highly virulent vEy-37 virus resulted in earlier rapid increase of IFN-α/β levels in the serum. Taken together, these data demonstrate that N^pro^ mediates suppression of IFN-α/β induction at the local replication sites in vivo, confirming predictions from cell culture data. In addition, these data support the model of CSF pathogenesis in which local IFN-α/β inhibition by N^pro^ contributes to enhanced virus replication resulting in elevated systemic IFN-α production and disease.

## Discussion

In the present study, restoring the zinc-binding domain with the N136D substitution in N^pro^ of the GPE^-^-derived viruses was sufficient to recover the capacity of N^pro^ to suppress IFN-α/β production completely in cell culture and at the local replication sites in vivo. Moreover, pigs inoculated with the virus carrying functional N^pro^ in the triple mutant virus (vGPE^-^/N136D; T830A; V2475A; A2563V) showed a longer course of viremia than pigs infected with the parental END^-^ triple mutant virus. Several reports suggested a role for the IRF3-degrading function of N^pro^ in persistent infection in vascular endothelial cells [26], and in mononuclear cells and lymphocytes of lymph node cells, splenocytes and gut-associated lymphoid tissue cells [27,28]. Meyers et al. suggested that N^pro^ and E^rns^ of bovine viral diarrhea virus are involved in establishing persistence after trans-placental infection in vivo [29]. All this strongly suggests that IRF3 degradation mediated by N^pro^ contributes to the establishment of persistent infections in vivo.

N^pro^-mediated suppression of IFN-α/β induction affected viral replication in vivo. Virus recovery from collected organs of pigs inoculated with the N^pro^ mutant and parental viruses were significant different between vGPE^-^/N136D; T830A; V2475A; A2563V and vGPE^-^/T830A; V2475A; A2563V infection on day 14 pi. Viremia was detected earlier after infection with viruses carrying the N^pro^ mutation. These data indicated that functional N^pro^ suppressed IFN-α/β induction and enhanced virus replication in tissues at the local replication sites, allowing the virus to spread more efficiently. CSFV pathogenicity was enhanced by restoring N^pro^ function in the moderately virulent virus backbone, whereas functional N^pro^ in the avirulent virus backbone did not confer any pathogenic phenotype. These results indicate that functional N^pro^ is not a virulence determinant on its own but acts as a co-factor of virulence by enhancing virus replication and spread through local inhibition of innate immune activation.

The presence of functional N^pro^ altered the production of IFN-α/β in infected pigs. Functional N^pro^ did significantly reduce the local IFN-α mRNA expression in the infected tissues while it enhanced the quantities of IFN-α/β in the circulation. The sera from the animals infected with the vGPE^-^/N136D; T830A; V2475A; A2563V virus had significantly higher IFN-α/β bioactivity than the sera from the vGPE^-^/T830A; V2475A; A2563V–infected pigs. The difference between local type I IFN inhibition and systemic type I IFN levels may be explained with the following hypothesis. The pDCs are the major producer of IFN-α/β during CSFV infection [30] and rely essentially on IRF7 for IFN-α induction [31]. In contrast, the non-pDC such as macrophages, conventional dendritic cells, endothelial cells and epithelial cells which are the major replication sites targeted by the virus induce type I IFN mainly through activation of IRF3 [32]. While in pDCs IRF7 is expressed constitutively, non-pDCs signal through IRF7 only after IRF7 induction by the IFN-dependent autocrine–paracrine amplification loop [33]. IRF7 is the most potent transcription factor for IFN-α production [34], and wild-type CSFV induces IFN-α synthesis more efficiently in pDCs compared with CSFV devoid of functional N^pro^[6]. Previous data demonstrated that functional N^pro^ inhibits the IRF3 pathway completely while it does only slightly reduce the activity of IRF7-dependent pathways [6,7]. Thus, in the different tissues representing the major replication sites of the virus, the GPE^-^-derived viruses with restored N^pro^ function suppress IFN-α/β production by blocking the IRF3 pathway, supporting local replication. The GPE^-^-derived virus that carries a functional N^pro^ and the triple mutations conferring virulence, as well as the highly virulent vEy-37 virus can then spread and replicate to high titers and infect pDC resulting in high systemic IFN-α levels.

In summary, this study demonstrates that the viral protein N^pro^ influences immune response in vivo and is required for enhanced pathogenicity. Understanding the molecular mechanisms involved in CSF pathogenesis, including persistent infections, will allow designing effective biological tools for controlling CSF.

## Competing interests

The authors declare that they have no competing interests.

## Authors’ contributions

TT carried out all experiments, contributed to data collection and analysis, and participated in drafting the manuscript; NN participated in the design of the study and conceived the qPCR experiment; NR participated in data analysis and drafting the manuscript; AS and HK participated in conceiving the study and drafting the manuscript, and YS participated in the design of the study, carried out the data analysis, conceived the experiment and prepared the manuscript. All authors read and approved the final manuscript.
